# Therapeutic potential of Liuwei Dihuang pill against KDM7A and Wnt/β-catenin signaling pathway in diabetic nephropathy-related osteoporosis

**DOI:** 10.1042/BSR20201778

**Published:** 2020-09-18

**Authors:** Ming Ming Liu, Rui Dong, Zhen Hua, Nan Ning Lv, Yong Ma, Gui Cheng Huang, Jian Cheng, Hai Yan Xu

**Affiliations:** 1 Department of Orthopedic Surgery, Lianyungang Second People’s Hospital, Jiang Su, China; 2 Institute of Traumatology and Orthopedics, Nanjing University of Chinese Medicine, Jiang Su, China; 3 Department of Orthopaedic Surgery, The First Affiliated Hospital of Zhejiang Chinese Medical University, Zhejiang, China; 4 Department of Orthopedics, Xuzhou Central Hospital, Jiang Su, China; 5 Xuzhou Clinical School, Xuzhou Medical University, Jiang Su, China; 6 Department of human anatomy, Xuzhou Medical University, Jiang Su, China

**Keywords:** Diabetic Nephropathy, KDM7A, Liuwei Dihuang Pill, MC3T3-E1 cells, Osteoporosis, Wnt/β-catenin Pathway

## Abstract

The effects of Liuwei Dihuang pill (LWDH) on diabetic nephropathy-related osteoporosis (DNOP) are unclear. The present study aimed to evaluate the effects of LWDH on KDM7A and Wnt/β-catenin signaling pathway in DNOP rats and the high glucose-induced MC3T3-E1 cells. A DNOP model was prepared by streptozotocin in 9-week-old male Sprague-Dawley (SD) rats to evaluate the effects of LWDH. The cell viability and differentiation capacity of high glucose-induced MC3T3-E1 cells were determined by CCK-8 assay, Alizarin Red staining, and alkaline phosphatase (ALP) staining, respectively. Furthermore, the expressions of KDM7A and Wnt1/β-catenin pathway-related proteins were determined by Western blot analysis. Treatment of DNOP rats with LWDH could significantly ameliorate the general state, degradation of renal function, and renal pathological changes. LWDH decreased the levels of TNF-α, IL-6, IL-8, IL-1β, ALP, and TRAP, and increased the calcium, phosphorus in serum, as well as decreased the level of the calcium and phosphorus in the urine. Besides, LWDH significantly improved bone mineral density (BMD), bone volume (BV), and the bone microstructure of DNOP rats. Moreover, LWDH increased the levels of the elastic modulus, ultimate load, and bending strength in the femurs. In MC3T3-E1 cells, serum-containing LWDH significantly increases in cell viability and osteoblastic differentiation capability. The expression of α-SMA, vimentin, KDM7A, Wnt1 and β-catenin were significantly down-regulated, and the E-cadherin, H3K9-Me2, H3K27-Me2, BMP-4, BMP-7, Runx2, osteocalcin, and Col1a1 were significantly up-regulated with LWDH treatment. The present study shows that LWDH has a therapeutic effect on DNOP, in part, through down-regulation of KDM7A and Wnt/β-catenin pathway.

## Introduction

Diabetes mellitus (DM) is a widespread metabolic disorder disease, characterized by insulin resistance and a constant decrease in insulin secretion, and it is expected to rise to 592 million by 2035 that threatening human health [1,2]. Diabetic nephropathy (DN), one of the most common long-term microvascular complications of DM, is a primary cause of the end-stage renal disease (ESRD), which occurs in approximately 20–40% of all DM patients [3,4]. Accumulating evidence shows that the imbalance of bone metabolism is significantly related to the state of hyperglycemia in DM, which finally results in osteoporosis [5]. Osteoporosis (OP) is one of the most common metabolic bone disorders characterized by low bone mass and damage of bone microarchitecture, and leads to increased risk and susceptibility of fragility fracture [6]. With the increasing prevalence of DM, osteoporosis is induced by DM, also known as diabetic osteoporosis (DOP), which is causing more pains for DM patients. Besides, the relevant clinical and experimental trials show that the low bone mass, decreased bone strength, and bone mineral density (BMD) are related to hyperglycemia, calcium, and phosphorus metabolic disorders in DN [7–9]. In addition, inflammation is a critical mechanism responsible for DOP [10]. Although many medical scholars made a lot of effort on diabetic nephropathy-related osteoporosis (DNOP); however, the exact pathogenesis of OP in DN is still unclear. And, therefore, it is urgent to investigate the underlying mechanisms and explore feasible drugs to improve the severities of DNOP.

In China, various herbs in Traditional Chinese medicine (TCM) have been widely used for the treatment of diseases for centuries, including DM, its complications, and postmenopausal osteoporosis (PMOP). Liuwei Dihuang (LWDH) pills are a famous traditional Chinese herbal medicine formula, and consist of six herbs (Rehmanniae *Radix Praeparata, Corni Fructus, Moutan Cortex, Dioscoreae Rhizoma, Poria*, and *Alismatis Rhizoma*), which developed in the book of “*Xiaoer YaoZheng ZhiJue”* by Qian Yi in the Song Dynasty. Zhao et al. demonstrated that LWDH is beneficial to diabetic microvascular complications such as diabetic retinopathy and diabetic nephropathy [4]. Also, it has been reported that the therapeutic effects of LWDH may be associated with the up-regulation of cardiotrophin-like cytokine factor 1 (CLCF1) and activation of the Janus kinase/signal transducer and activator of transcription (JAK/STAT) pathway in PMOP [11]. However, the effects and mechanism of LWDH on OP of diabetic DN were not clear.

Histone lysine(K)-specific demethylase 7 (KDM7) subfamily has drawn more attention as a transcriptional co-activator that composed of three members: KDM7A, KDM7B, and KDM7C [12]. KDM7A, also known as JHDM1D, is a member of the plant homeodomain (PHD) finger protein (PHF) family of PHD-and JmjC domain-containing histone demethylases. Recent studies demonstrate that the KDM7A is a dual demethylase for H3K9m2 and H3K27m2 and regulates the CCAAT/enhancer binding protein α (C/EBPα) and fibroblast growth factor 4 (FGF4) expression to promote the osteogenic and neural differentiation [12,13]. Additionally, KDM7A also was significantly up-regulated in prostate cancer tissue [14], and the silencing of KDM7A significantly inhibited breast tumor growth *in vivo* [15]. Up to now, it remains unknown whether KDM7A is associated with the development of DNOP. Meanwhile, many studies have demonstrated that the universal pathologic feature of DN mostly associated with the glomerular basement membrane thickening, mesangial expansion, and accumulation of extracellular matrix (ECM), as well as accompanied by continuous albuminuria [3,16]. Particularly, renal interstitial fibrosis is the prominent biological event of DN, and the renal tubular epithelial-to-mesenchymal transition (EMT), characterized by the increased expressions of the mesenchymal markers, vimentin, α-smooth muscle actin (α-SMA), and the decreased expression of the epithelial marker E-cadherin in tubular epithelia cell, is a relatively complex pathological mechanism on the DN. On the other hand, recent studies have proved that the canonical Wnt/β-catenin signaling pathway plays a significant role in the progression of osteoporosis [17,18]. Aberrant Wnt also contributes to the onset of diabetes [19]. β-Catenin acts as the master regulator of osteoblastic proliferation and differentiation [20]. Additionally, accumulating studies illustrated that the Wnt/β-catenin signaling pathway aid in the prevention of inflammation on osteoblast differentiation [21,22]. Further, evidence has demonstrated that sophisticated cross-talk between KDM7A and Wnt/β-catenin in osteogenic differentiation [11,23]. To investigate the role of the LWDH pills in DNOP, STZ-induced DN rats and high glucose-induced MC3T3-E1 cells were established. In the present study, we found that LWDH could attenuate DN-induced osteoporosis by inhibiting inflammation and EMT, which was significantly dependent on the blockage of KDM7A and Wnt/β-catenin signaling pathway. Thus, our finding suggested that LWDH could be served as a promising candidate to ameliorate DNOP progression.

## Materials and methods

### Animals and treatments

Ninety 4-week-old male Sprague-Dawley (SD) rats (63–68 g in weight) were purchased from Shanghai Silaike Laboratory Animal Company (Shanghai, China; Certification No: No. SCXK2007-0005), and were raised in the Animal Center of Zhejiang Chinese medical university (Zhejiang, China; Certificate No. SYXK (ZHE) 2018-0012). All rats were housed in a 12-h light/dark cycle at 23 ± 1°C with free access to water and standard chow diet. Following acclimation for 1 week, in 90 SD rats, we randomly have chosen 10 rats as a control group and fed with regular chow, and another 80 rats were used for the establishment of DN models. The model group was feeding with a high-fat diet, including 71.5% basal feed, 10% lard, 10% egg yolk, 5% glucose, 2.5% milk powder, and 1% cholesterol for 4 weeks. Then, after fasting 12 h, the rats were intraperitoneally injected with freshly prepared streptozotocin (STZ, 65 mg/kg, Sigma-Aldrich) in sodium citrate buffer (pH 4.5) to induce DN. An equal volume of citrate buffer (0.1 M) was injected into control rats. After 3-day STZ injections, the rats with random blood glucose level higher than 16.7 mmol/l were considered to be diabetic animals. Subsequently, these DM rats were continually fed a fat diet for 3 weeks, and had urinary albumin (ALB) ≥ 30 mg /24 h, indicating the successful establishment of the DN model. After that, 8 weeks later, the BMD also was assessed by using dual-energy X-ray absorptiometry (DXA) (InAlyzer, MEDIKORS Inc., Seoul, Korea). If the BMD level was markedly decreased, the rat could be confirmed as a DNOP rat. Finally, 50 DNOP rats were randomly divided into five groups, namely DNOP group (*n*=10), LWDH low dose-treated group (*n*=10), LWDH medium dose-treated group (*n*=10), LWDH high dose-treated group (*n*=10), and alendronate (ALN) sodium-treated group (positive control, *n*=10). Rats in the low-, medium-, and high-dose LWDH groups received 1.8, 3.6, or 5.4 g LWDH (Beijing Tong Ren Tang Co., Ltd. Beijing, China) /kg of body weight, respectively, using an oral gavage once daily for 12 weeks. Rats in the ALN group and the control and DNOP groups received an equal volume of drinking water or 70 μg ALN (Yangtze River Pharmaceutical Group, Taizhou, Jiangsu, China) /kg of body weight/ week, respectively. The rats in each group were weighed weekly. All animal procedures were approved by the animal care and welfare committee of Zhejiang Chinese Medical University (permit number: ZSLL-2019-104, Zhejiang, China).

### Dual-energy X-ray absorptiometry

After 12 weeks of LWDH administration, the whole body and the left proximal femurs were scanned using Dual-Energy X-ray Absorptiometry (DXA) before sacrifice. BMD and bone volume (BV) were measured using the InAlyzer software (Seongnam, Korea).

### Determination of biochemical parameters

After X-ray testing, all rats were moved into the metabolic cages to measure 24-h urine volume. Then, the rats were killed under chloral hydrate anesthesia (300 mg/kg, IP), and the blood was collected from the heart and centrifuged at 3000 rpm for 15 min at 4°C to obtain the serum. Serum and urine samples were stored at −80°C for further studies. Subsequently, the kidneys were aseptically removed out of the rats, rinsed with cold isotonic saline, and weighed. At the end of the experiments, euthanasia occurred under chloral hydrate anesthesia followed by cardiac puncture and kidneys removal for all animals. The left renal tissues were used for histopathological examination, and the right renal tissues were put in liquid nitrogen and stored at −80°C for subsequent assays. Besides, the kidney index (the ratio of kidney weight/body weight) was calculated. Finally, the left femurs were collected for biomechanical testing, Western blot analysis, and stored in 10% formaldehyde for micro-CT analysis. Serum levels of fasting blood glucose, creatinine, blood urea nitrogen (BUN), aspartate aminotransferase (AST), alanine aminotransferase (ALT), and urinary ALB, as well as creatinine, were quantified using a Hitachi 7080 Chemistry Analyzer (Hitachi, Ltd., Tokyo, Japan).

### The Hematoxylin/Eosin (H&E) staining

The kidney tissues were fixed in 4% paraformaldehyde solution (Sigma, U.S.A.), and embedded in paraffin. Paraffin sections (4-μm thickness) were cut and stained with hematoxylin & eosin (H&E). Images of the kidney sections were captured using a light microscope (Olympus BX53, Tokyo, Japan) at a magnification of ×200 and ×400.

### Determination of inflammation and bone metabolism parameters

Serum level of inflammation marker TNF-α, IL-6, IL-8, IL-1β, Alkaline phosphatase (ALP), tartrate resistant acid phosphatase (TRAP), calcium, and phosphorus, as well as urine level of calcium and phosphorus were measured using ELISA kits (Nanjing Jiancheng Bioengineering Institute) based on the manufacturer’s instructions.

### Micro-computed tomography (CT) analysis

The microarchitecture of the left femurs was investigated with a μCT scanner with 12 μm voxel size (Model LaTheta LCT-200; Hitachi-Aloka, Tokyo, Japan), using the following settings: 300 µA tube current, 75 kV tube voltage, 0.7 degree rotational step, 75 ms exposure time, and 2 frames averaged in each step. The 3D microarchitecture of the proximal segment of the femur was re-established to set as the region of interest (ROI), and then analyzed with the LaTheta software (version 3.20). Then, the μCT quantitative parameters were automatically calculated within the ROI with the help of the analyzing software such as bone mineral contents (BMC), trabecular number (Tb.N), trabecular separation (Tb.Sp), trabecular thickness (Tb.Th), connectivity density, bone volume/tissue volume (BV/TV), and structure model index (SMI).

### Biomechanical testing

To evaluate the biomechanical properties of femurs after μCT scanning, a three-point bending assay was performed by using an electronic universal testing machine (Model RGWF4005, Shenzhen Reger Instrument Co. Ltd., China). In brief, the femur was fixed two supporting points, then a load was vertically added to the femoral midshaft until it is fractured. The load at the breaking point was identified as the ultimate load. And the elastic modulus, bending strength of the femurs were also recorded.

### Preparation of medicated sera

Another 40 male SD rats weighing 220–280 g were also purchased from Shanghai Silaike Laboratory Animal Company (Shanghai, China), and were housed in the standardized conditions. The experiment was approved by the animal care and welfare committee of Zhejiang Chinese Medical University (permit number: ZSLL-2019-106, Zhejiang, China). Animals were randomly divided into the low- (*n*=10), medium- (*n*=10), and high- (*n*=10) dose LWDH, and normal control (*n*=10) groups. The low-, medium-, and high- dose LWDH group rats were administered intragastrically LWDH at the dose of 1.8, 3.6, and 5.4 g/kg of body weight, respectively, twice a day for 5 days. Normal control animals were administered intragastrically saline on the same schedule. Two hours after the final administration, blood was collected from the abdominal aorta under sodium pentobarbital anesthesia (150 mg/kg, Sigma, U.S.A.) and centrifuged. Finally, the serum samples of each group were filtered through a 0.22 μm filter membrane, heat-inactivated at 56°C for 30 min, and stored at −80°C until further use.

### Cell culture

MC3T3-E1 cells were purchased from American Type Culture Collection (ATCC®CRL-2594; Rockville, MD, U.S.A.). MC3T3-E1 cells were grown in low-glucose α-MEM medium supplemented with 10% fetal bovine serum (FBS), 100 U/ml penicillin, and 100 μg/ml streptomycin at 37°C in 5% CO_2_ atmosphere with a relative humidity of 85–95%, and the medium was subsequently changed every 2–3 days.

### Cell viability assay

The MC3T3-E1 cells were stimulated with high glucose for 14 days and then placed into 96-well plate at 5 × 10^3^ cells/well in 200 μl medium. Following serum starvation for 2 h, 10% FBS and 10% normal rats serum (NRS) were, respectively, added to the culture medium for 24 h. The serum-free (SF) cells were added with sterile PBS and acted as the blank control. Besides, to assess the effect of LWDH-medicated serum on MC3T3-E1 cell viability, LWDH sera (at concentrations of 2.5, 5, and 10%) were added to the culture medium for 24 h. Subsequently, 10 μl CCK-8 reagent was added to each hole and incubated for 3 h under a dark condition in the incubator. Finally, the absorbance (OD value) was read at 450 nm by using a microplate reader.

### Osteoblastic differentiation assay

After induction of high glucose for 14 days, MC3T3-E1 cells were seeded into a 24-well plate at 1 × 10^4^/ml in the low-glucose α-MEM supplemented with 10% serum that consists of 2.5% LWDH-medicated serum+7.5% NRS, 5% LWDH-medicated serum+5% NRS, or 10% LWDH-medicated serum, respectively, containing 10^−8^ mol/l dexamethasone, 50 mg/l ascorbic acid, and 10 mmol/l β-glycerophosphate (Sigma, U.S.A.), and was used as the calcification medium. The cells were handled with 10% NRS and they were considered as the control group. The culture medium was discarded every 48 h and cultured for 25 days. Then, Alizarin Red staining and ALP staining were performed to detect the calcium nodules, and granule or block deposit, respectively. Images of cells were captured under an inverted phase-contrast microscope.

### Western blotting assay

The femoral heads, kidney tissue, and cell homogenates were prepared by extracting proteins by using RIPA lysis buffer (Solarbio, Beijing, China) containing protease inhibitors, phenylmethanesulfonyl fluoride, and phosphatase inhibitor. After quantization using a BCA protein assay kit (Beyotime), the protein samples (30 μg) were separated by 8%, 10% or 12% SDS-PAGE and transferred to a polyvinylidene difluoride (PVDF) membrane and blocked in 5% BSA 1 h at room temperature. The membranes were incubated with primary antibodies [α-SMA (1:2000,ab32575, Abcam); Vimentin (1:1000, ab92547, Abcam); E-cadherin (1:1000, ab76055, Abcam); KDM7A (1:2000, A14692, ABclonal, Wuhan, China); H3K9-Me2 (1:1000, A2359, ABclonal, Wuhan, China); H3K27-Me2 (1:1000, A2362, ABclonal, Wuhan, China); Wnt1 (1:2000, ab63934, Abcam); β-catenin (1:1000, ab16051, Abcam); BMP4 (1:2000; ab39973, Abcam); BMP7 (1:2000, ab56023, Abcam); Runx2 (1:1000, ab76956, Abcam); Osteocalcin (OCN, 1:2000, ab13420, Abcam); Collagen 1 (Colla1, 1:3000, ab34710, Abcam); β-actin (1:1000, ab8227, Abcam); GAPDH (1:2500, ab9485, Abcam); and Histone H3 (1:500, ab1220, Abcam)] at 4°C overnight. After washing with TBST, the membranes were further probed with appropriate horseradish peroxidase-conjugated secondary antibodies in TBST for 2 h at room temperature. Labeled protein bands were visualized using a high sensitivity ECL luminous liquid and the images were captured with Azure Bioimaging systems (California, U.S.A.). The bands were quantitatively determined using Image-J software (NIH, Bethesda, MD, U.S.A.).

### Statistical analysis

The results are represented as mean ± standard deviation (SD). Statistical analyses were performed using the SPSS v22.0 (IBM, Armonk, NY, U.S.A.). Normal distribution was analyzed with the Shapiro–Wilk test. Statistical differences were evaluated by one-way analysis of variance (ANOVA) followed by Tukey’s post-hoc tests. *P* < 0.05 was considered statistically significant.

## Results

### Effect of LWDH on the general features of DNOP rats

**Figure 1:**
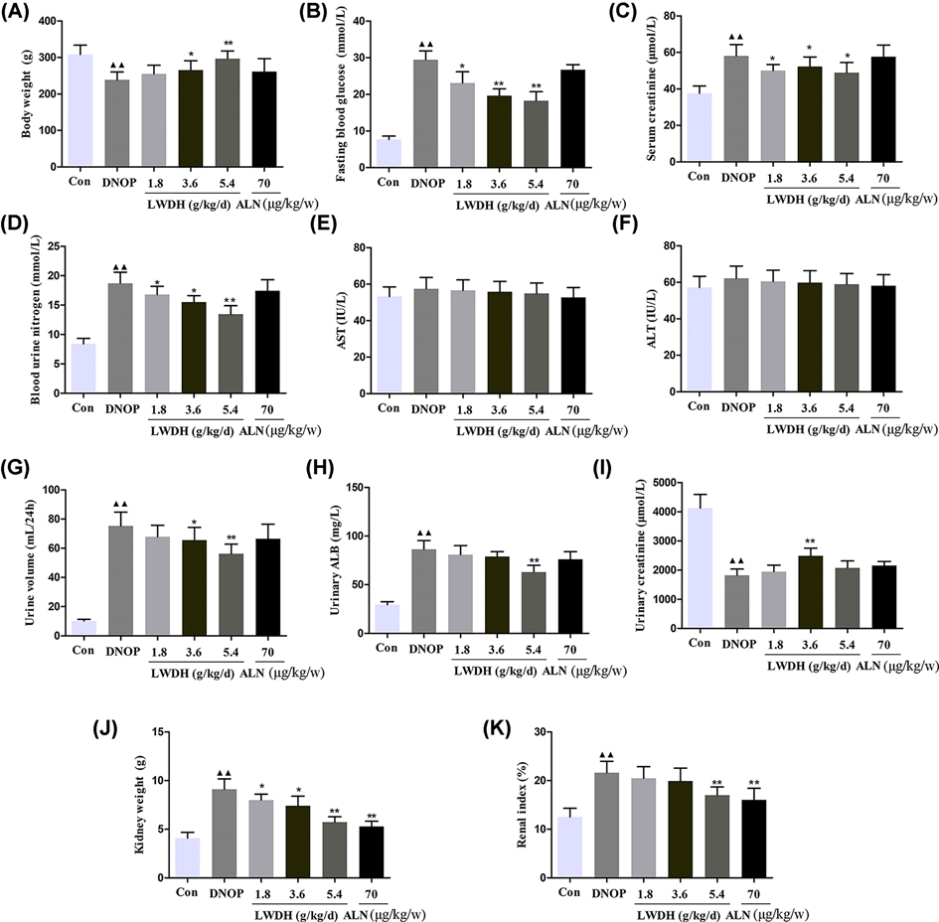
Effect of LWDH on body weight, blood glucose, and renal function markers in DNOP rats (**A**) Body weight, (**B**) fasting blood glucose, (**C**) serum concentrations of creatinine, (**D**) blood urine nitrogen, (**E**) AST, (**F**) ALT, (**G**) urine volume, (**H**) urinary ALB, (**I**) urinary creatinine, (**J**) kidney weight, and (**K**) renal index. All data were expressed as the mean ± SD (*n*=10). ^▲▲^*P*<0.01 compared with the control group; *P<0.05, **P<0.01 compared with the DNOP group.

**Figure 2:**
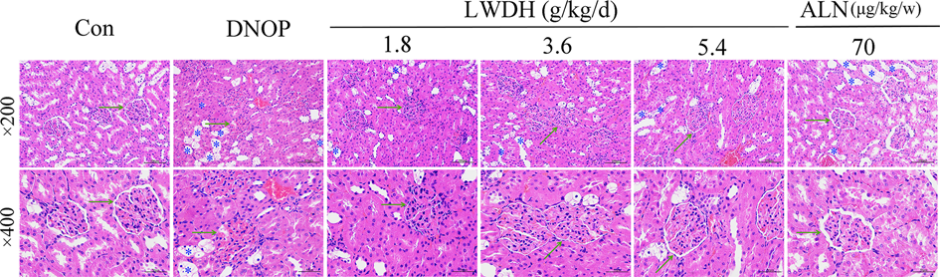
Effects of LWDH on renal histopathological changes in DNOP rats Representative photographs of renal sections after stained with hematoxylin–eosin. Glomerular hypertrophy, basement membrane (GBM) thickening, and necrotic and dilated tubules were clearly observed in the DNOP group. After the administration of LWDH, the above changes were reduced. Green arrow pointing to the glomerulus. Yellow asterisk indicates dilated or necrotic tubules (magnification, × 400, scale bar = 50 μm; magnification, × 200, scale bar = 100 μm).

After administration of LWDH for 12 weeks, the body weighs of the rats in the high and medium dose LWDH groups showed a significant increase (Figure 1A), and decrease in fasting blood glucose, serum creatinine, and BUN level compared with those of rats in the DNOP group (Figure 1B–D). In addition, AST and ALT levels had no difference among the six groups (Figure 1E,F). Compared with the DNOP group, we found that the high dose-treated group exhibited lower levels of urine volume and urinary ALB (Figure 1G,H), respectively, while the level of urinary creatinine was elevated in the medium dose-treated group (Figure 1I). Furthermore, as shown in Figure 1J,K, in comparison with the DNOP group, the level of kidney weight and, renal index were lower in the high dose- and ALN- treated groups. Next, kidney tissues were also collected for HE staining, and the representative pathological change of HE staining is displayed in Figure 2. HE staining revealed normal glomerular and tubular structures in the control group. However, glomerular hypertrophy, basement membrane (GBM) thickening, renal interstitial inflammatory cell infiltration, and necrotic and dilated tubules were clearly observed in the DNOP group. The pathological changes were improved after LWDH treatment in OP rats with DN to a certain extent. These results indicate that LWDH has the ability to improve renal dysfunction in the absence of hepatic and renal toxicity.

### Effect of LWDH on the EMT associated markers of kidney tissue in DNOP rats

**Figure 3:**
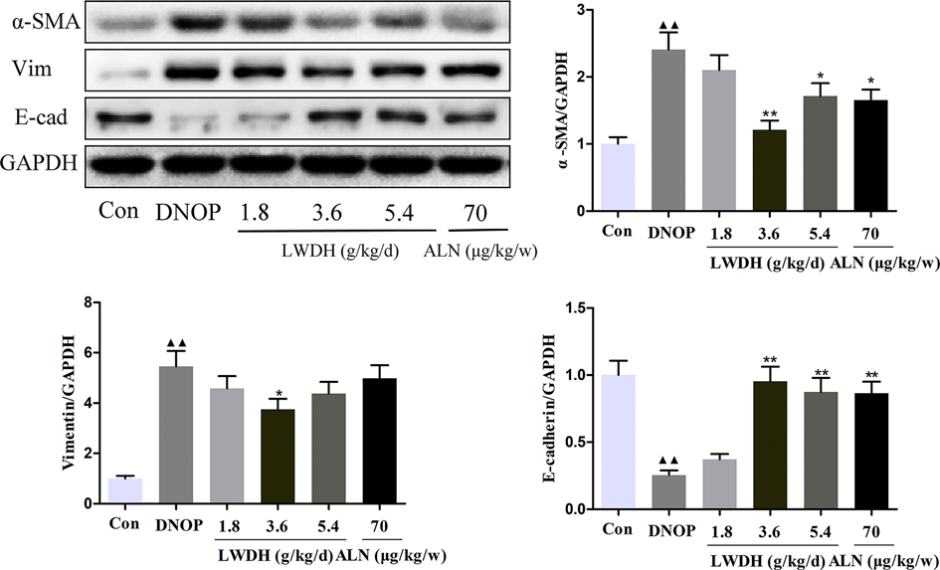
Effects of LWDH on the epithelial-to-mesenchymal transition of kidney tissue in DNOP rats Western blot images and relative expression of α-SMA, Vimentin (Vim), and E-cadherin (E-cad). All data are expressed as the mean ± SD (*n*=3). ^▲▲^*P*<0.01 compared with the control group; *P<0.05, **P<0.01 compared with the DNOP group.

Accumulating studies have revealed that multiple pathological damages were associated with the progression of DN, including EMT, so we determined the expression of EMT associated proteins, including, α-SMA, Vimentin (Vim), and E-cadherin (E-cad). Compared with in control group, the expression of α-SMA, and Vim was increased in the DNOP group and decreased in the high dose LWDH group; E-cad was down-regulated in the DNOP group and up-regulated in the high and medium dose LWDH and ALN groups in the present study (Figure 3 and Supplementary Figure S1). These results suggested that the reno-protective effects of LWDH possibly through the regulation of the EMT process.

### Effect of LWDH on the inflammatory mediators in DNOP rats

**Figure 4:**
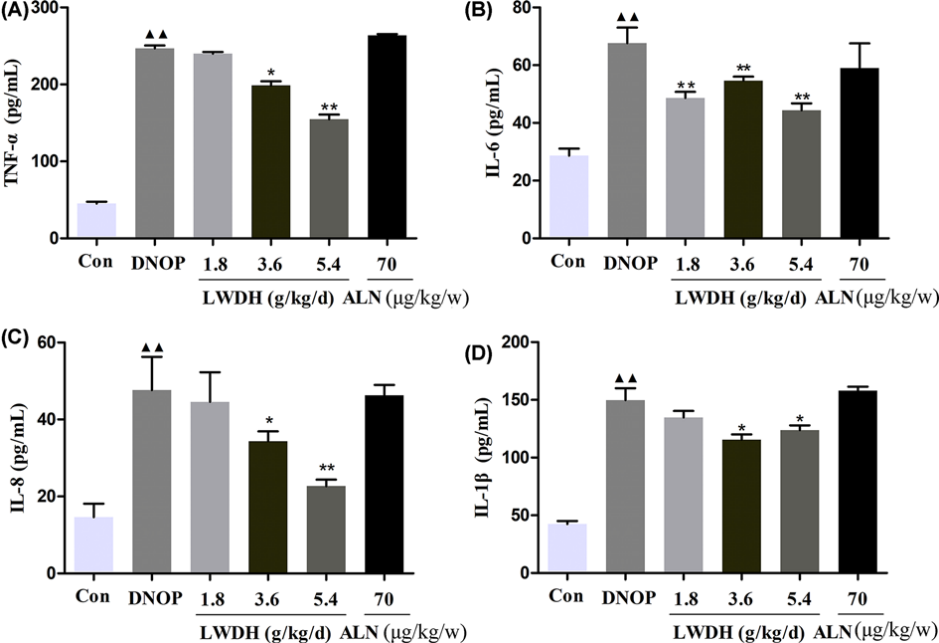
Effect of LWDH on serum (**A**) TNF-α, (**B**) IL-6, (**C**) IL-8, and (**D**) IL-1β levels in DNOP rats The data are expressed as the means ± SD (*n* =10); ^▲▲^*P*<0.01 compared with the control group; *P<0.05, **P<0.01 compared with the DNOP group.

DOP rats are confirmed associated with the inflammation, and multiple cytokines such as IL-6 and IL-1β, are closely related to the bone resorption. In the present study, we further investigated whether LWDH could ameliorate the inflammatory markers in DNOP rats. As shown in Figure 4A–D, compared with the DNOP model group, high and medium dose LWDH groups showed significantly decreased TNF-α, IL-6, IL-8, and IL-1β levels. Additionally, DNOP rats treated with ALN showed not differ compared with the model group on the inflammatory marker levels. Collectively, these results suggested that treatment with LWDH may reduce the inflammation in DNOP rats to a certain extent.

### Effect of LWDH on the bone metabolism in DNOP rats

**Figure 5:**
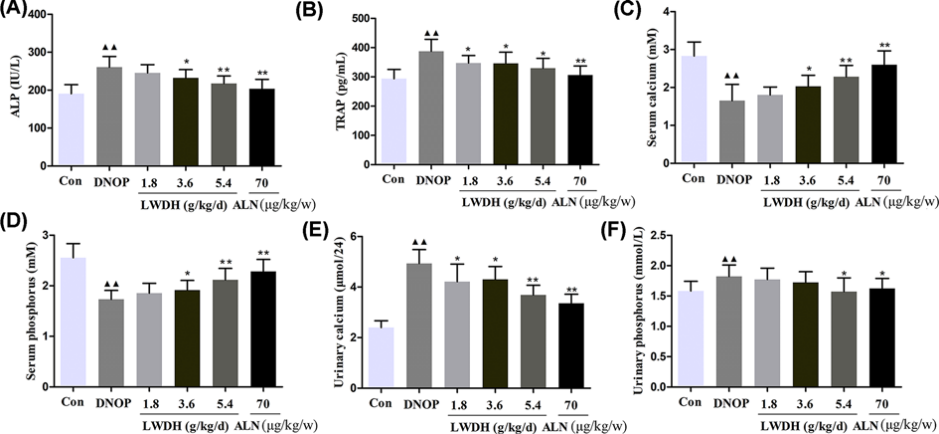
Effect of LWDH on bone metabolism-related biomarker in DNOP rats Serum concentrations of ALP (**A**), TRAP (**B**), calcium (**C**), phosphorus (**D**), as well as urine concentrations of calcium (**E**), phosphorus (**F**) in each group were investigated by using commercial ELISA kits. Data are presented as mean ± SD (*n*=10). ^▲▲^*P*<0.01 compared with the control group; *P<0.05, **P<0.01 compared with the DNOP group.

The bone metabolism-related blood markers are shown in Figure 5. The serum biomarker ALP content levels, an important indicator of bone formation and the serum bone-specific TRAP levels, an important indicator of bone resorption, were significantly decreased at any dose of LWDH group and ALN group (Figure 5A,B). Further, significantly decreased serum calcium and phosphorus levels in the DNOP rats were up-regulated in the rats after being treated with LWDH and ALN, respectively (Figure 5C,D). Also, the content of the urinary calcium and phosphorus were measured. Compared with the DNOP group, the high dose LWDH group and ALN group had decreased urinary calcium and phosphorus (Figure 5E,F). Taken together, these results illustrated that LWDH may ameliorate bone metabolism in DNOP rats under the experimental condition.

### Effect of LWDH on the bone density and microstructure in DNOP rats

**Figure 6:**
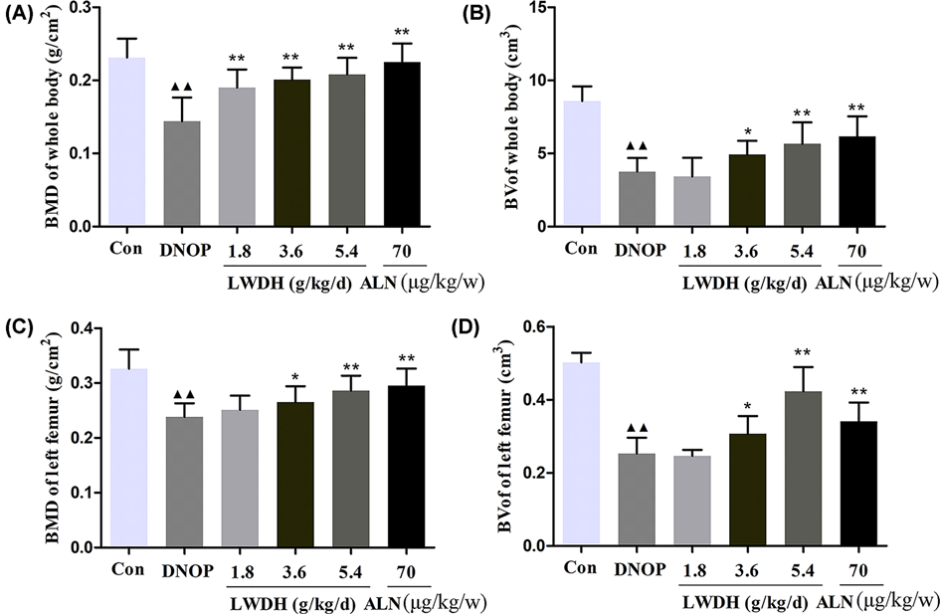
Effect of LWDH on (A) bone mineral density (BMD), and (B) bone volume (BV) for the whole body, and (C) BMD, and (D) BV for the left femur Values are the mean ± SD (*n*=10). ^▲▲^*P*<0.01 compared with the control group; *P<0.05, **P<0.01 compared with the DNOP group.

**Figure 7:**
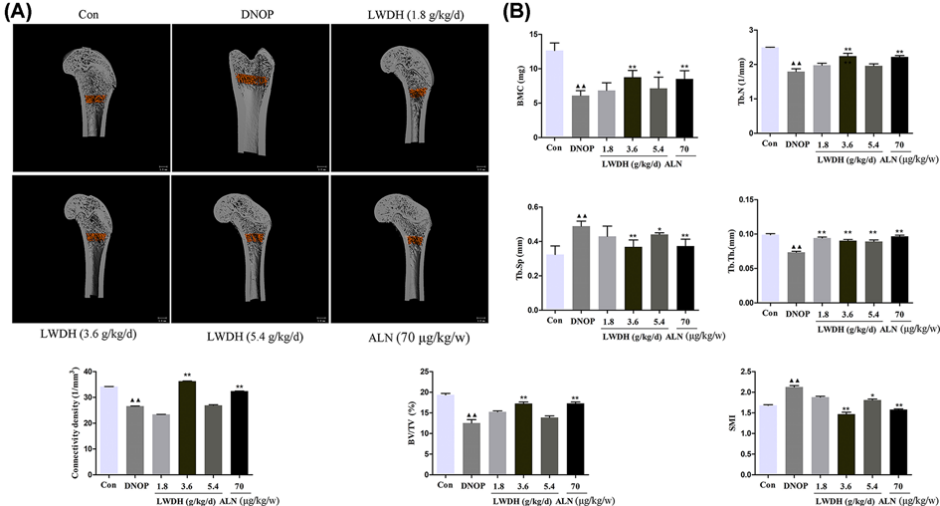
Effect of LWDH on bone microstructure in DNOP rats (**A**) Representative micro-CT images of the distal femoral trabecular bone. (**B**) Bone microarchitecture of BMC, Tb.N, Tb.Sp, Tb.Th, connectivity density, BV/TV, and SMI. All data are expressed as the mean ± SD (*n*=10). ^▲▲^*P*<0.01 compared with the control group; *P<0.05, **P<0.01 compared with the DNOP group.

To assess the bone density and microstructure among the experimental groups, the BMD and BV of the whole body and left femur, respectively, were detected by DXA. As shown in Figure 6A,B, compared to the BMD and BV of the DNOP group, BMD and BV were significantly increased, respectively, in any dose of LWDH and ALN groups. Moreover, the results of BMD and BV of the left femur showed fairly similar trends o those in the whole body (Figure 6C,D). In addition, micro CT analyses were employed to assess the difference between the trabecular bone microarchitecture in DNOP rats. As shown in Figure 7A, the results of representative micro-CT images suggested that LWDH could observably inhibit the trabecular bone mass loss and significantly attenuated the degeneration of trabecular bone microarchitecture. LWDH treatment evidently improved femoral BMC levels compared to DNOP groups; Tb.N, Tb.Th, connectivity density, and BV/TV decreased in the DNOP group, were significantly increased in the LWDH-/ALN-treated group; Additionally, augment in Tb.Sp and SMI femur was observed while being reduced by LWDH and ALN (Figure 7B).

### Effect of LWDH on the femoral biomechanical properties in DNOP rats

**Figure 8:**
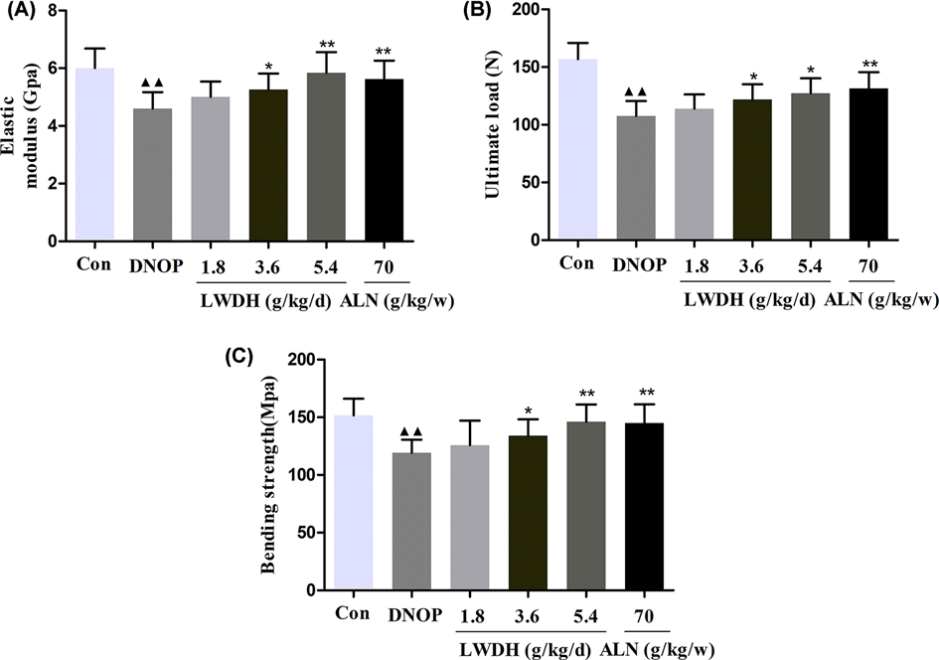
Effect of LWDH on biomechanical properties in DNOP rats (**A**) The elastic modulus, (**B**) ultimate load, and (**C**) bending strength in each group were measured by the three-point bending test. Data are presented as mean ± SD (*n*=10). ^▲▲^*P*<0.01 compared with the control group; *P<0.05, **P<0.01 compared with the DNOP group.

To further test the potential role of LWDH on the bone biomechanical properties, the rat femurs were measured through a three-point bending assay. As shown in Figure 8A–C, the elastic modulus, ultimate load, and bending strength were significantly decreased compared with that detected in the DNOP group. Notably, treatment with LWDH and ALN significantly increased these biomechanical parameters in rat femurs compared with the DNOP rats. These results indicate that LWDH might be a promising strategy to promote bone strength, microstructure, and bone biomechanical properties in DNOP rats.

### Effect of LWDH on the KDM7A, Wnt/β-catenin signaling, and differentiation in the bone of DNOP rats

**Figure 9:**
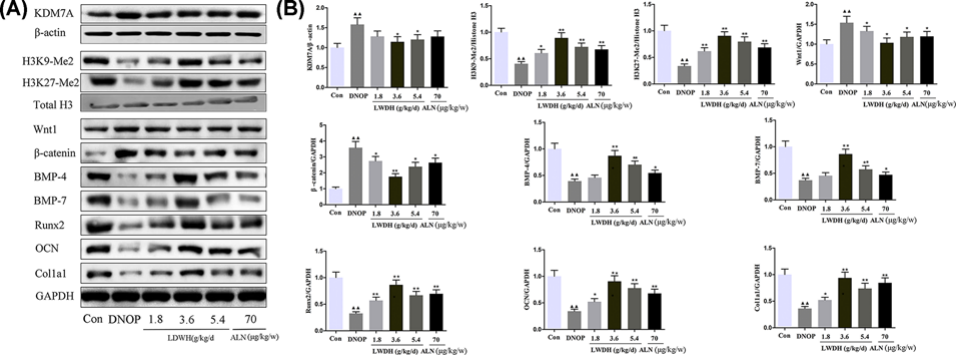
Effect of LWDH on KDM7A, Wnt1/β-catenin signaling, and osteoblast differentiation-related proteins expression of the femur tissue in DNOP rats (**A and B**) Western blot analysis for KDM7A, H3K9-Me2, H3K27-Me2, Wnt1, β-catenin, BMP-4, BMP-7, Runx2, OCN, and Col1a1 expression in the femur tissue of DNOP rats. Data are presented as mean ± SD (*n*=6). ^▲▲^*P*<0.01 compared with the control group; *P<0.05, **P<0.01 compared with the DNOP group.

To find out the effects of LWDH on inhibiting KDM7A, Wnt/β-catenin signaling pathway, and differentiation in the rat femurs, the protein levels of KDM7A, H3K9-Me2, H3K27-Me2, Wnt1, β-catenin, BMP-4, BMP-7, Runx2, OCN, and Col1a1 were examined through western blot assay. As illustrated in Figure 9A,B (Supplementary Figure S2), we found that the expression of KDM7A in the rat femurs of DNOP rats was significantly increased compared with the control group. LWDH treatment significantly attenuated the elevated expression of KDM7A. Knowing that KDM7A has the histone demethylase activity, so we investigated the histone methylation in bone tissue. The expression levels of H3K9-Me2 and H3K27-Me2 in the DNOP group were decreased compared with the control group. Interestingly, LWDH and ALN administration ameliorated this effect. Additionally, LWDH and ALN also reduced the expression of Wnt1 and β-catenin. Furthermore, the expression of osteoblast differentiation-related proteins, BMP-4, BMP-7, Runx2, OCN, and Col1a1, were markedly increased in the LWDH or ALN- treated DNOP rats. Together, the results suggested that LWDH may through inhibiting the KDM7A and Wnt/β-catenin signaling expressions to improve osteoblast differentiation of bone tissue in the DNOP rats.

### Effect of LWDH-medicated serum on cell viability and osteoblastic differentiation of MC3T3-E1 cells

**Figure 10:**
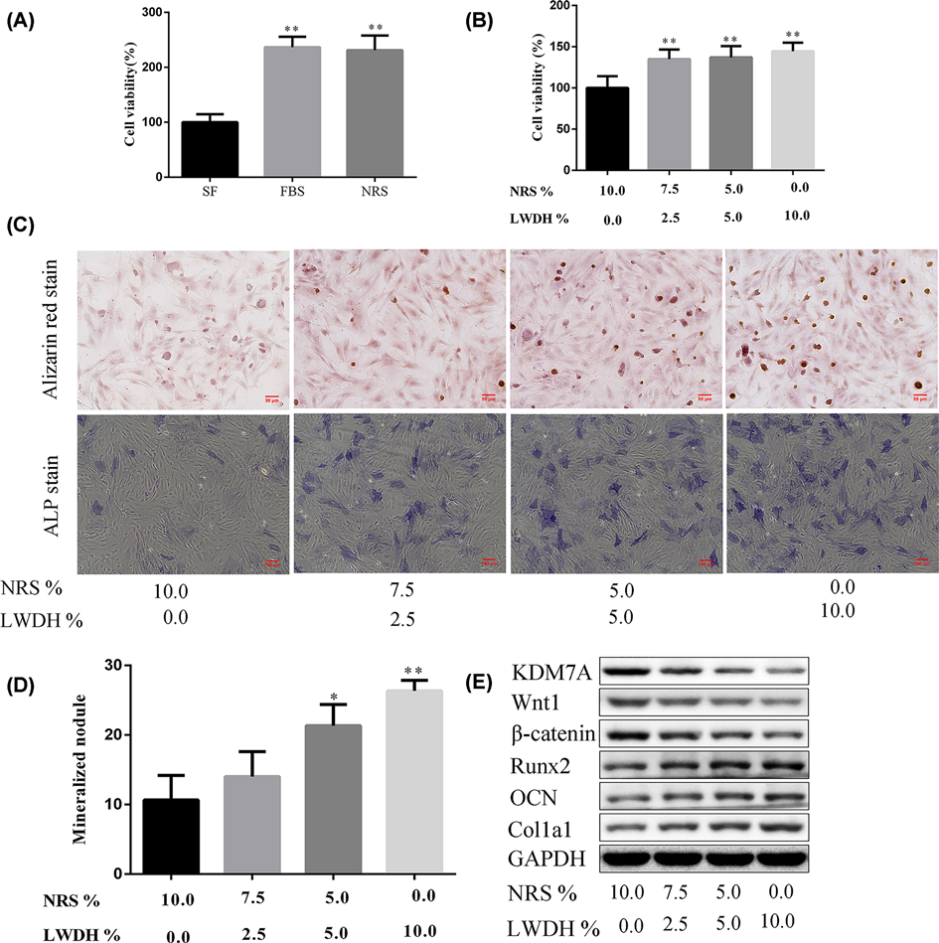
Effect of LWDH-medicated serum on osteoblastic differentiation of MC3T3-E1 cells (**A**) The effect of NRS on cell viability of MC3T3-E1 cells. (**B**) Cell viability of high glucose-induced MC3T3-E1 cells in cultures with 2.5% LWDH-medicated serum+7.5% NRS, 5% LWDH-medicated serum+5% NRS, or 10% LWDH-medicated serum, respectively. (**C**) The differentiation of MC3T3-E1 cells was observed by Alizarin Red staining (scale bar = 50 µm) and ALP staining (scale bar = 100 µm). (**D**) Semi-quantitative analysis of mineralized nodule in Alizarin Red staining. (**E**) Representative Western blot images of KDM7A, Wnt1, β-catenin, Runx2, OCN, and Colla1 in high glucose-induced MC3T3-E1 cells that were treated with LWDH-medicated serum. GAPDH served as an internal control.

As shown in Figure 10A, the result of CCK-8 assay showed that the cell viability of MC3T3-E1 cells with incubation of 10% FBS and 10% NRS, respectively, was significantly improved when compared with the SF-treated cells. And, there is no statistical difference in cell viability between FBS and NRS group; so, the NRS was used as the substrate to prepare the different concentrations of LWDH-medicated serum, and the blank control in the subsequent experiments. Besides, in the present study, the high-dose LWDH-medicated serum was primarily used to investigate the osteoblastic differentiation of MC3T3-E1 cells, because high-dose LWDH has a significant improvement of osteoporosis in the DNOP rats. As expected, with the increasing LWDH-medicated serum concentrations (2.5%, 5%, 10% LWDH), the cell viability of high glucose-induced MC3T3-E1 cells was gradually increased (Figure 10B). Alizarin Red staining showed that the typical orange calcified nodules were shaped, and the mineralized nodule was apparently increased in a concentration-dependent manner. Also, ALP staining showed that the cytoplasm has a positive reaction of gray-black granule or more block deposit with the increase of LWDH-medicated serum (Figure 10C,D). To further explore whether the osteoblastic differentiation effect of LWDH was associated with the inhibition of KDM7A, Wnt1/β-catenin signaling, Western blotting was used to examine the expression of KDM7A, Wnt1, β-catenin, and osteoblast differentiation-related proteins of MC3T3-E1 cells following the exposure of high glucose and calcification medium with LWDH-medicated serum administration. Figure 10E (Supplementary Figure S3) depicts that LWDH-medicated serum attenuated the expression KDM7A, Wnt1, and β-catenin, and there was an effective increase in the levels of Runx2, OCN, and Colla1 in MC3T3-E1 cells.

## Discussion

Diabetic nephropathy (DN) and diabetic osteoporosis are becoming the most important issues globally. Therefore, impeding their development and progression, as well as discovering novel drugs are still need further investigation. With this aim, in the present study, we explored whether Liuwei Dihuang pills (LWDH), exhibited a promising protection role in diabetic nephropathy-related osteoporosis (DNOP). Here, we found that LWDH showed a significant role in reducing serum fasting blood glucose, creatinine, blood urine nitrogen, urine volume, urinary ALB, urinary creatinine, and the renal index levels in DNOP rats. In addition, morphological observation of kidney tissue can effectively reveal the pathological condition of DN, including glomerular hypertrophy, GBM thickening, infiltration of inflammatory cells, and ECM accumulation. Our results suggested that LWDH could significantly improve the depravation of DN.

It is established that EMT is a common and complicated pathological process observed in DN rats [24]. In the present study, LWDH treatment significantly decreased the expression of α-SMA and Vimentin levels and increased the level E-cadherin protein of rats after 12 weeks compared with the DNOP group. The modern pharmacological study showed that the relevant active ingredients of LWDH played the effective roles of anti-oxidation, anti-inflammation, and immune regulation [4,25]. In addition, it is reported that the inflammation helps in the pathogenesis of DN and osteoporosis [26–28]. Interestingly, treatment with LWDH effectively decreases the serum concentrations of TNF-α, IL-6, IL-8, and IL-1β in DNOP rats, which contributed to the improvement of diabetic osteoporosis. Accumulating evidence confirmed that bone remodeling plays a distinct role in regulating bone structure. The osteoblasts and osteoclasts are a participant in this physiological event for sustaining the balance of bone formation and resorption [29]. Previous *in vitro* study by Xia et al. reported that the LWDH-contained serum significantly increases ALP activity and amount of calcified nodules for promoting the differentiation of osteoblasts that isolated from neonatal rat calvariae [18]. It is well accepted that the serum specific biomarkers of bone formation (ALP) and bone resorption (TRAP) are widely selected to assess the bone remodeling [30]. Zheng et al. reported that a significantly increased serum ALP levels in the ovariectomized diabetic rats compared with the control [31], and Liu et al. found that a decrease in TRAP level in serum was observed in diabetic rats with *Mori Folium* treatment [32]. Intriguingly, we found that the up-regulation of ALP and TRAP were markedly alleviated by LWDH and alendronate (ALN) treatment. As previously described, calcium deficiency and/or calcium malabsorption were identified as one of the central factors in osteoporotic patients, and phosphorus is as important as calcium in supporting bone augmentation and maintenance [33,34]. The results from the present study indicated that LWDH and ALN significantly ameliorated the serum calcium and phosphorus levels, and decreased the concentration of urine calcium and phosphorus. Collectively, the inflammatory state and bone metabolism-related biochemical indicators in DNOP rats were significantly reversed by treatment with LWDH.

Dual-Energy X-ray Absorptiometry and micro-CT analysis of the present study found that LWDH and ALN presented bone protective roles in DNOP rats. LWDH administration significantly ameliorated the further severity of the femoral trabecular bone microstructure as well as improve bone material properties in DNOP rats, as shown by improved the BMD and BV of the whole body and the left femur, respectively; BMC, Tb.N, Tb.Th, connectivity density, and BV/TV, and reduced the Tb.Sp and SMI. Acevedo et al. demonstrated that the degeneration of bone microstructure and material properties may destroy the bone strength and increase the fracture risk [35]. In the present study, we noticed that treatment with LWDH and ALN for 12 weeks resulted in a significant increase in elastic modulus, ultimate load, and bending strength level, which is consistent with the results of the previous study [18,32]. Together, the current findings suggest that LWDH can improve bone quality in DNOP rats.

In our previous study, we found that catalpol enhanced the proliferation and differentiation of high glucose-induced MC3T3 cells by inhibiting the expression of KDM7A by activating the Wnt/β-catenin signaling pathway [23]. Besides, Huang et al. found that KDM7A as a novel histone demethylase was associated with the differentiation of neural differentiation [36]. Yang et al. revealed that KDM7A balances adipogenic and osteogenic differentiation from progenitor cells via the epigenetic control of Wnt signaling [12]. The Wnt/β-catenin pathway is known to be linked to DM, DN, and OP. Liu et al. suggesting that the *Rehmanniae Radix Preparata* attenuates bone loss and improves bone quality in ovariectomized rats by interfering with the Wnt/β-catenin signaling pathway [37]. Jiating et al. reported metformin promotes osteogenic differentiation of osteoblasts through the inhibition of the GSK3β/Wnt/β-catenin pathway [21]. These literatures are further supported by the fact that the DNOP can be improved by depressing the KDM7A and Wnt/β-catenin pathway. Additionally, Runx2, a prominent target of the Wnt/β-catenin pathway, is essential for the differentiation of osteoblasts [38]. On the other hand, the BMP signaling pathway also plays a significant role in osteoblast differentiation by increasing Runx2 expression [39]. The present study demonstrated that the expressions of KDM7A, Wnt1, and β-catenin were down-regulated, and the expressions of H3K9-Me2 and H3K27-Me2 were up-regulated in the femurs of DNOP rats following supplement of LWDH, as well as up-regulation of the osteoblast proteins such as BMP-4, BMP-7, Runx2, OCN, and Col1a1 expressions in DNOP rats.

In the present study, we further demonstrate that LWDH-medicated serum increased the cell viability of high glucose-induced MC3T3-E1 cells. In addition, treatment with LWDH-medicated serum aids in increasing the formation of calcified nodules and enhancing ALP activity in the MC3T3-E1 cells. Furthermore, the present study demonstrated the protein expression levels of KDM7A, Wnt1, and β-catenin were obviously decreased, and significantly increased Runx2, OCN, and Col1a1 levels in the high glucose-induced MC3T3-E1 cells following the administration of LWDH-medicated serum. Taken together, we supposed that LWDH treatment attenuated DNOP and promoted osteogenic differentiation might be through KDM7A and Wnt/β-catenin signaling inhibition, subsequently inhibiting inflammation. Nevertheless, there are still shortcomings in the present study. The quality control of the LWDH pill should be evaluated through HPLC, and future studies are still necessary to elucidate the effects of LWDH on DM complications, which will contribute to clarify the mechanisms underlying for diabetic osteoporosis.

## Conclusions

In conclusion, these results show that Liuwei Dihuang pill is beneficial to diabetic nephropathy-related osteoporosis by suppressing inflammation, in part, through down-regulation of KDM7A and Wnt/β-catenin signaling pathway.

## Supplementary Material

Supplementary Figures S1-S3

## Data Availability

The data of this study available from the corresponding author on reasonable request.

## Abbreviations

ALN: alendronate
ALP: alkaline phosphatase
BMD: bone mineral density
BV: bone volume
DNOP: diabetic nephropathy-related osteoporosis
EMT: epithelial-to-mesenchymal transition
GBM: glomerular hypertrophy, basement membrane
LWDH: Liuwei Dihuang pill
SD: Sprague-Dawley
