# Supplementary material for: Therapeutic potential of Liuwei Dihuang pill against KDM7A and Wnt/β-catenin signaling pathway in diabetic nephropathy-related osteoporosis
Source: Biosci Rep. 2020 Sep 18;40(9):BSR20201778. doi: 10.1042/BSR20201778 (PMC7502694; doi:10.1042/BSR20201778)
Supplement: Supplementary Figures S1-S3 [file BSR-2020-1778_supp.pdf]

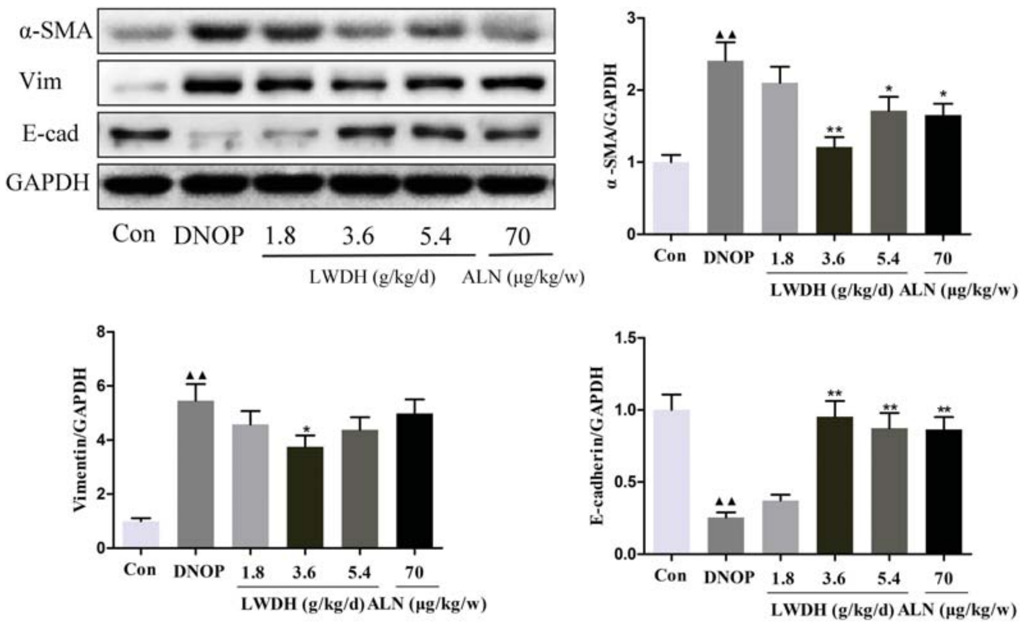

Figure 3 Effects of LWDH on the epithelial-to-mesenchymal transition of kidney tissue in DNOP rats. Western blot images and relative expression of α-SMA, Vimentin (Vim), and E-cadherin (E-cad). All data are expressed as the mean ± SD (n = 3). ▲▲*P<0.01* compared with the control group; **P<0.05*, ***P<0.01* compared with the DNOP group.

supplementary file Figure S1

Full unedited versions of the Western blots for Figure 3

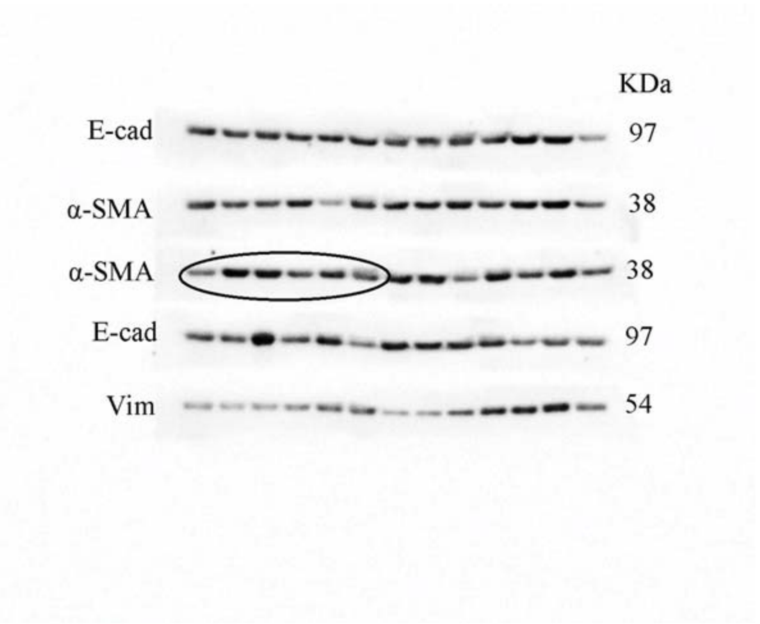

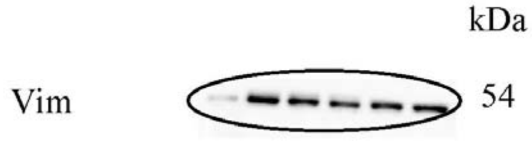
kDa
Vim
54

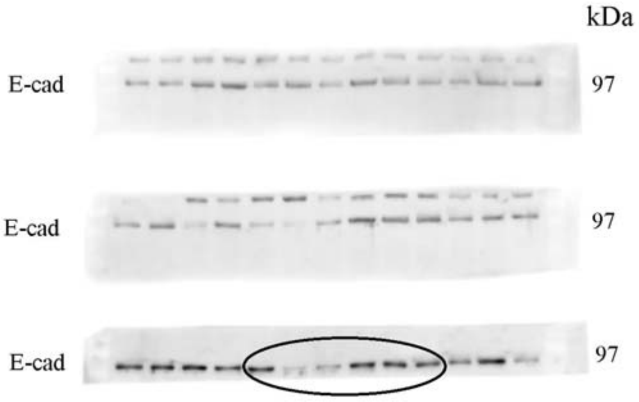
kDa
E-cad
97
E-cad
97
E-cad
97

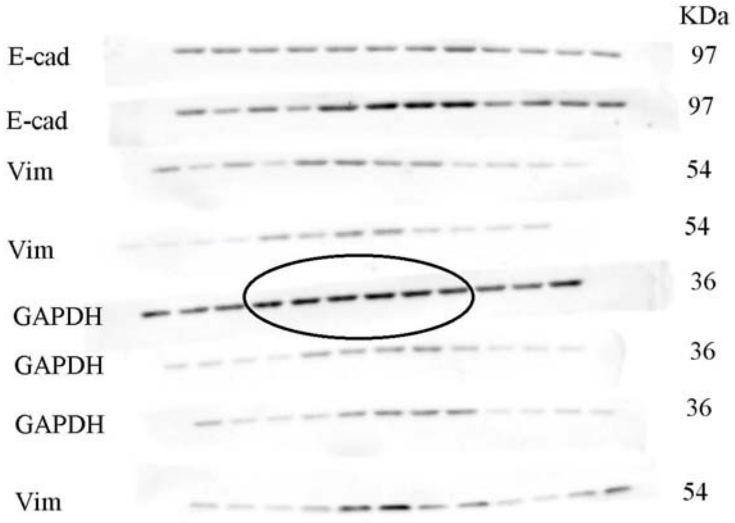
KDa
E-cad
97
E-cad
97
Vim
54
Vim
54
GAPDH
36
GAPDH
36
GAPDH
36
Vim
54

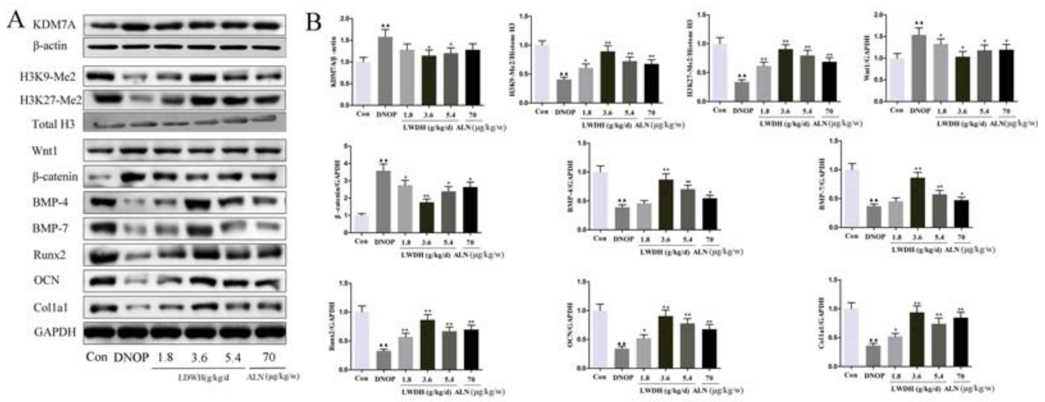

Figure 9 Effect of LWDH on KDM7A, Wnt1/β-catenin signaling, and osteoblast differentiation-related proteins expression of the femur tissue in DNOP rats. (A, B) Western blot analysis for KDM7A, H3K9-Me2, H3K27-Me2, Wnt1, β-catenin, BMP-4, BMP-7, Runx2, OCN, and Col1a1 expression in the femur tissue of DNOP rats. Data are presented as mean ± SD (n =6). ▲▲*P<0.01* compared with the control group; **P<0.05*, ***P<0.01* compared with the DNOP group.

supplementary file Figure S2

Full unedited versions of the Western blots for Figure 9

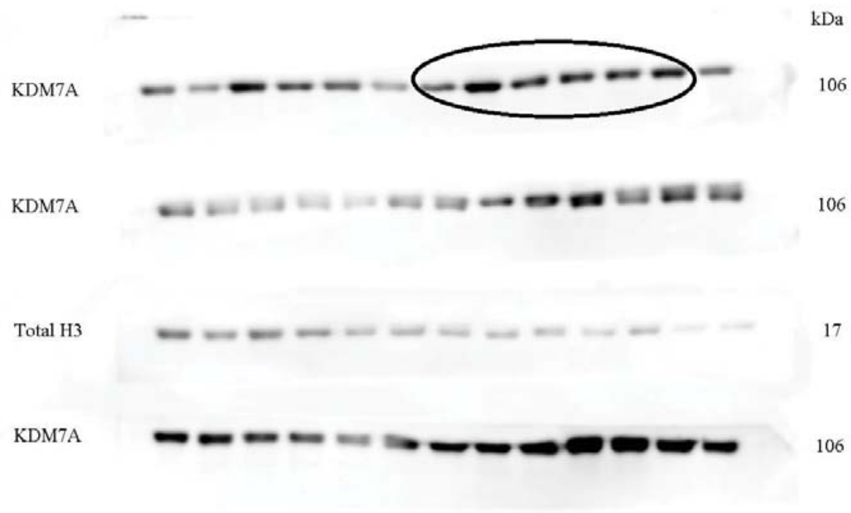

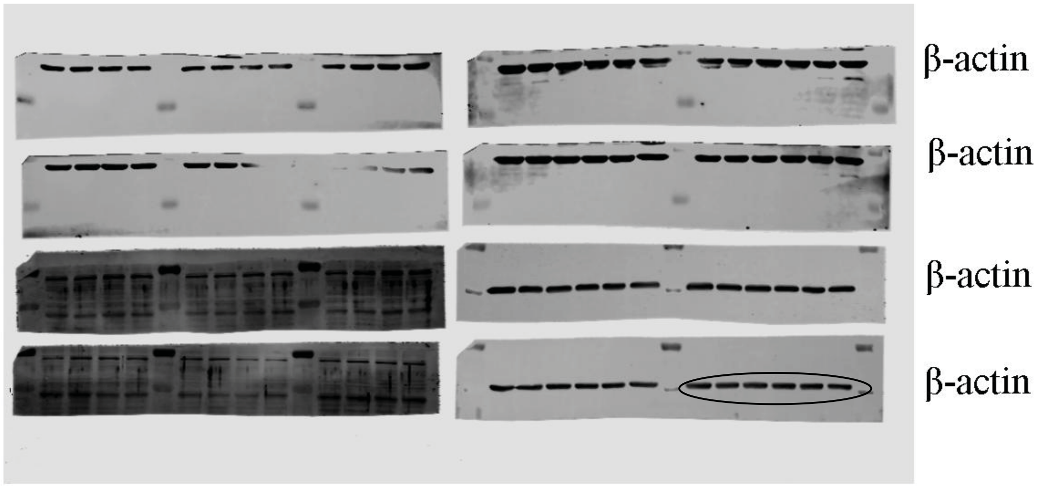
β-actin
β-actin
β-actin
β-actin

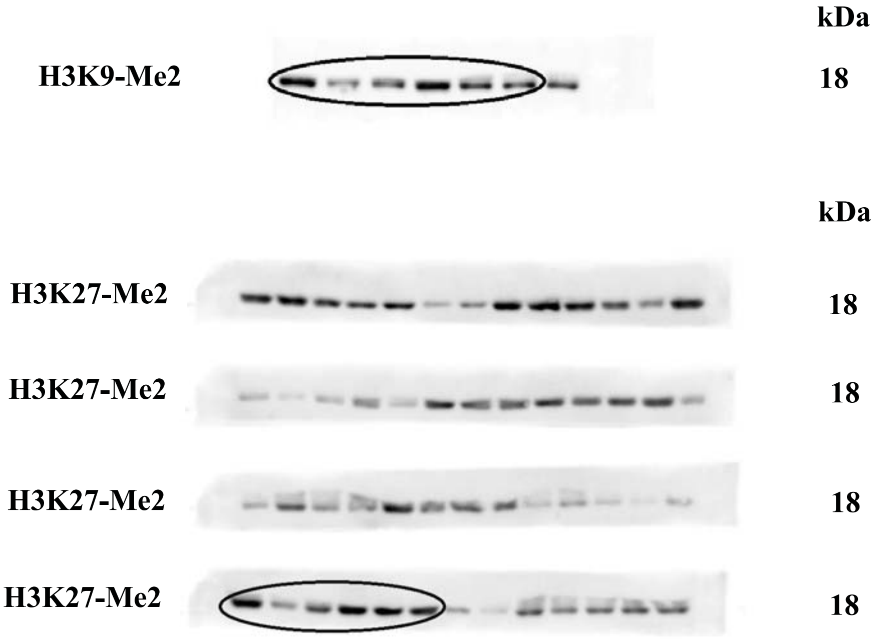
kDa
H3K9-Me2
18
kDa
H3K27-Me2
18
H3K27-Me2
18
H3K27-Me2
18
H3K27-Me2
18

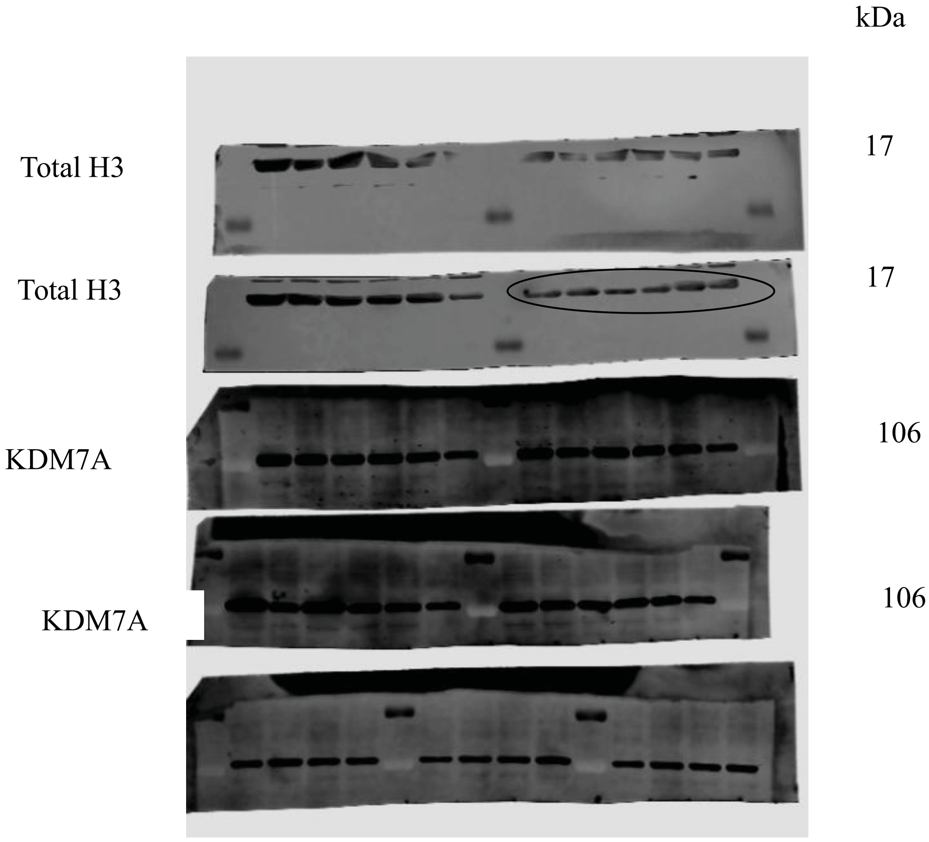
kDa
Total H3
17
Total H3
17
KDM7A
106
KDM7A
106

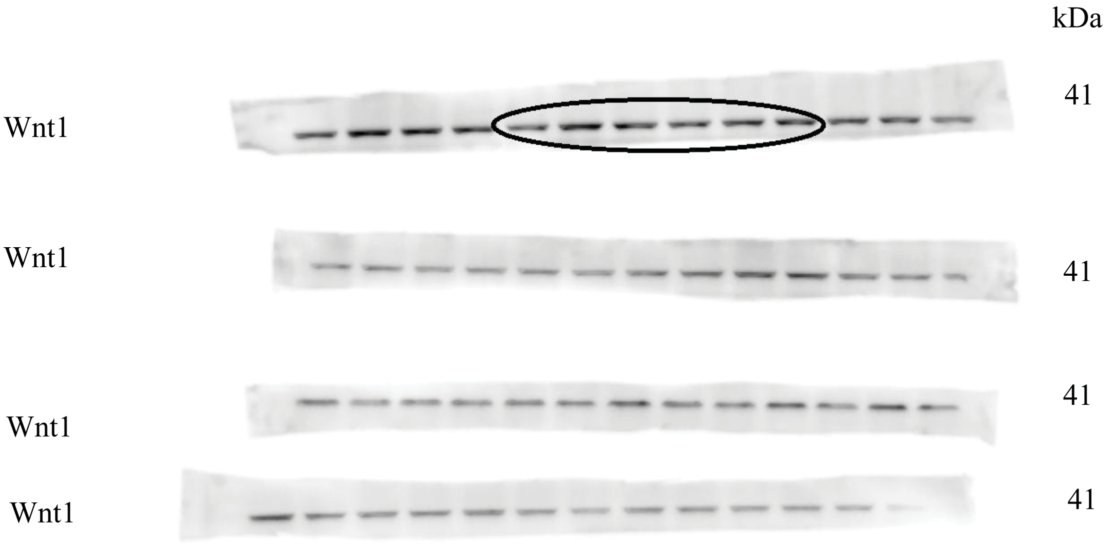
kDa
Wnt1
41
Wnt1
41
Wnt1
41
Wnt1
41

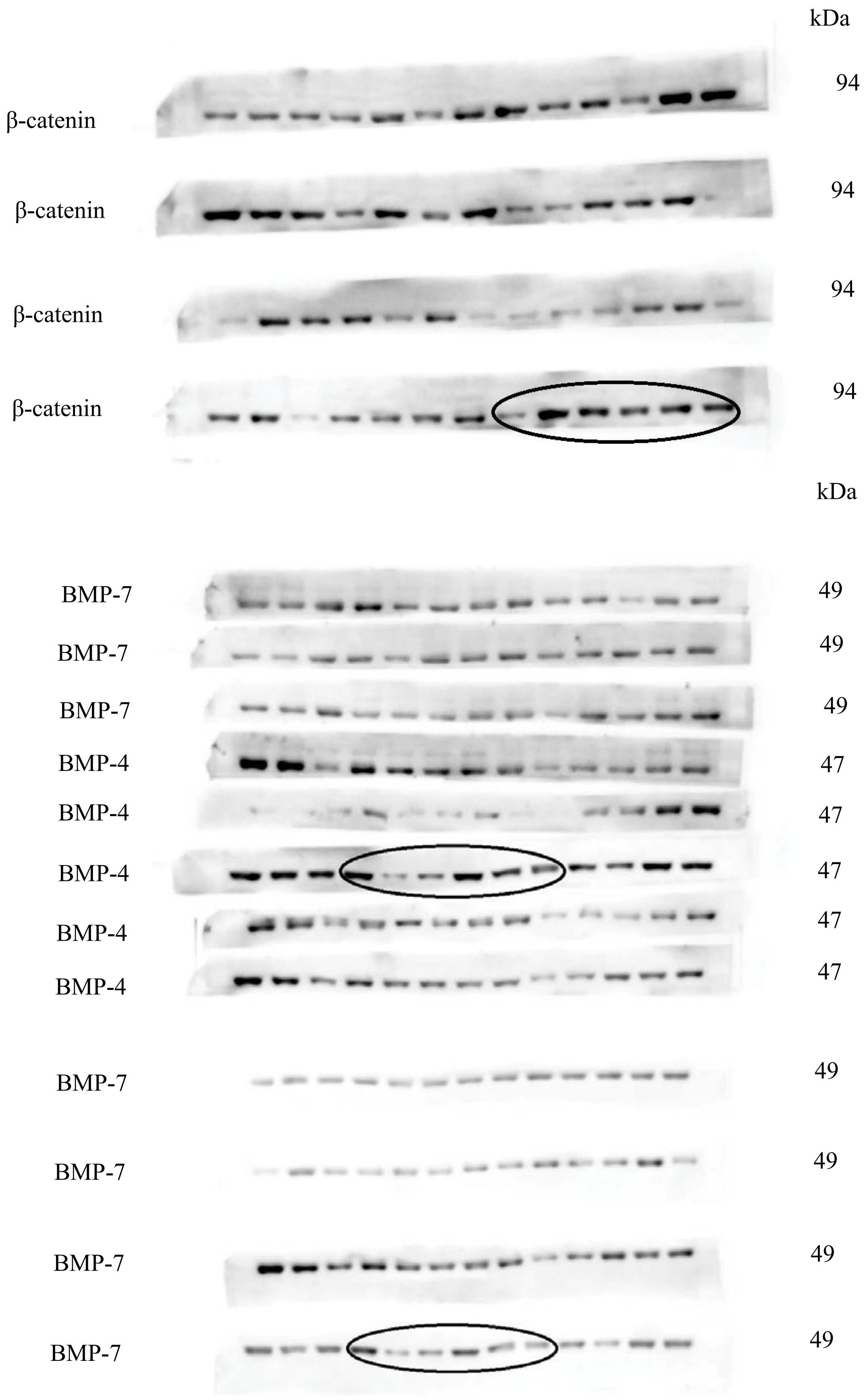
kDa
β-catenin
94
β-catenin
94
β-catenin
94
β-catenin
94
kDa
BMP-7
49
BMP-7
49
BMP-7
49
BMP-4
47
BMP-4
47
BMP-4
47
BMP-4
47
BMP-4
47
BMP-7
49
BMP-7
49
BMP-7
49
BMP-7
49

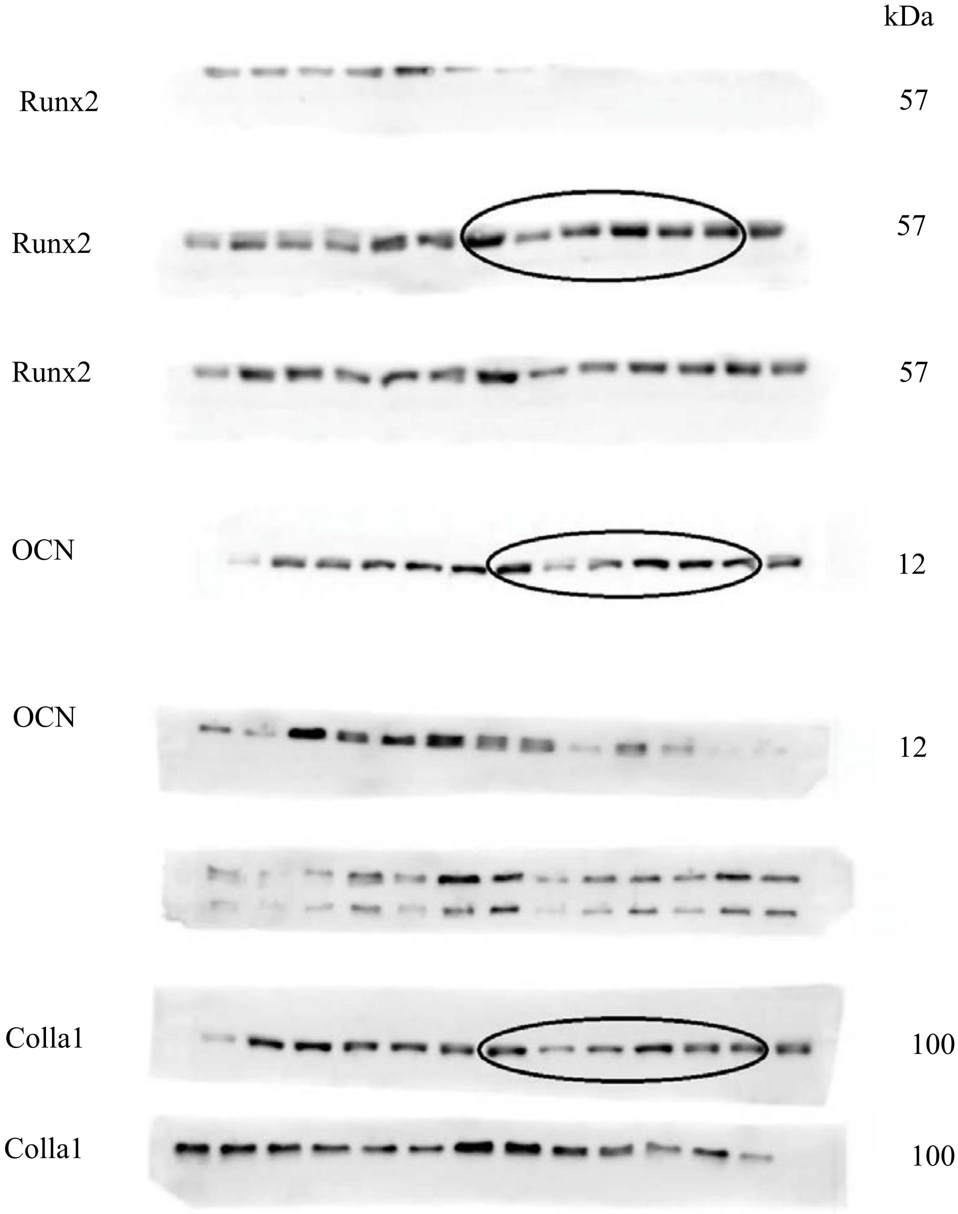
kDa
Runx2
57
Runx2
57
Runx2
57
OCN
12
OCN
12
Colla1
100
Colla1
100

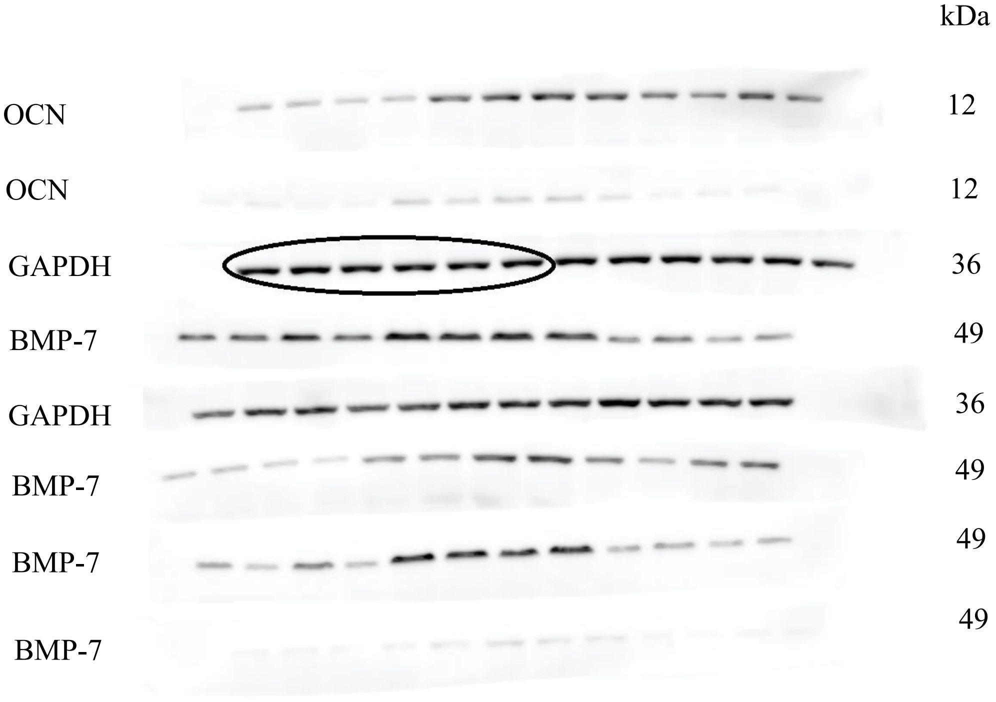
kDa
OCN
12
OCN
12
GAPDH
36
BMP-7
49
GAPDH
36
BMP-7
49
BMP-7
49
BMP-7
49
